# Early Neuroimmune Modulation in Hereditary Cerebellar Ataxias: Experimental Opportunities in Zebrafish Models

**DOI:** 10.3390/cells15111014

**Published:** 2026-05-31

**Authors:** Valentina Naef, Michela Giacich, Devid Damiani, Filippo Maria Santorelli

**Affiliations:** Neurobiology and Molecular Medicine Units, IRCCS Fondazione Stella Maris, 56128 Pisa, Italy; michela.giacich@fsm.unipi.it (M.G.); devid.damiani@fsm.unipi.it (D.D.); filippo.santorelli@fsm.unipi.it (F.M.S.)

**Keywords:** hereditary cerebellar ataxia, Purkinje cells, neuroinflammation, microglia, zebrafish model, flavonoids, naringenin

## Abstract

Hereditary cerebellar ataxias are progressive neurodegenerative disorders for which disease-modifying treatments remain lacking. Although these conditions have traditionally been investigated from a neuron-centered perspective, evidence from several ataxia models indicates that changes in the cerebellar immune microenvironment can arise before overt neuronal loss and may contribute to early circuit dysfunction. This review examines hereditary cerebellar ataxias through the lens of early neuroimmune regulation, with particular attention to the region-specific properties of cerebellar microglia and their roles in synaptic refinement, inflammatory tone modulation and circuit homeostasis. We further discuss zebrafish as a useful experimental system for this question, because they combine in vivo imaging, genetic manipulation, and scalable functional assays in an intact vertebrate model. In this context, flavonoids—and especially naringenin—are not considered as immediate therapeutic candidates, but as mechanistically informative experimental probes to investigate how modulation of neuroimmune signaling affects disease-relevant phenotypes in vivo. By integrating genetic ataxia models with dynamic neuroimmune readouts, functional behavioral assays, and circuit-level analyses, zebrafish-based approaches can help identify early windows during which neuroimmune signaling influences cerebellar resilience and disease progression and can guide subsequent validation in mammalian systems.

## 1. Introduction

Neurodegenerative disorders comprise a heterogeneous group of neurological conditions characterized by structural and functional disruption of neural networks, leading to progressive neuronal loss and ultimately resulting in severe motor, cognitive, and behavioral impairments [1]. Despite their clinical diversity, growing evidence indicates that neuroinflammation represents a common and early pathogenic component across a broad range of neurological conditions, including neurodegenerative diseases, psychiatric disorders, and brain injuries [2]. In the context of this review, the term “neuroinflammation” is used in a broad and contemporary sense to describe dynamic alterations of the local neuroimmune environment within the central nervous system, primarily referring to dynamic alterations of resident glial populations, including microglia and astrocytes, and their associated signaling pathways. Although classical neuroinflammatory responses may also include infiltration of peripheral immune cells under conditions of blood–brain barrier disruption, the present discussion mainly focuses on intrinsic neuroimmune signaling, glial reactivity, and inflammatory pathways that emerge within cerebellar circuits independently of overt peripheral immune invasion. Inflammation is a fundamental component of immune defense, encompassing coordinated biological processes that eliminate harmful stimuli, remove damaged tissue, and promote repair. However, persistent activation of innate immune responses, particularly involving microglia and astrocytes, can contribute to disease progression through chronic cytokine release, oxidative stress, and synaptic dysfunction, ultimately driving neuronal damage and network failure [2]. Among neurodegenerative conditions, hereditary ataxias provide a valuable framework for investigating the interaction between neuronal vulnerability and immune-mediated mechanisms. These disorders are primarily characterized by impaired motor coordination and cerebellar dysfunction. Hereditary cerebellar ataxias comprise a highly heterogeneous group of disorders, including polyglutamine expansion diseases, ion channel disorders, mitochondrial diseases, and defects affecting DNA repair or cellular proteostasis. Despite these diverse molecular etiologies, increasing evidence suggests convergence toward shared pathogenic mechanisms involving altered neuroimmune signaling, oxidative stress, and glia–neuron dysfunction within vulnerable cerebellar circuits [3,4,5]. Neuropathologically, these disorders are characterized by progressive degeneration of cerebellar circuits, particularly involving Purkinje cells, deep cerebellar nuclei, and, depending on the specific subtype, additional extracerebellar structures such as the brainstem, spinal cord, retina, or peripheral nerves. Clinically, patients commonly present with gait instability, impaired coordination, dysarthria, oculomotor abnormalities, and progressive motor dysfunction, often accompanied by cognitive, sensory, or systemic manifestations in more complex forms. Despite their marked genetic heterogeneity, many hereditary ataxias converge on shared mechanisms including mitochondrial dysfunction, impaired proteostasis, oxidative stress, and altered neuron–glia interactions, all of which may contribute to selective cerebellar vulnerability [3,4,5]. In this context, immune-mediated cerebellar ataxias provide an important conceptual precedent, demonstrating that cerebellar dysfunction can emerge from potentially reversible immune-driven mechanisms before irreversible Purkinje cell loss becomes established [6]. Although tremor may coexist in some cerebellar disorders, hereditary cerebellar ataxias are primarily characterized by progressive impairment of coordination and cerebellar circuit dysfunction rather than isolated tremor syndromes.

Although pathogenic proteins are often broadly expressed throughout the brain, the basis of selective cerebellar vulnerability remains incompletely understood. Increasing evidence supports a contributory role of neuroimmune mechanisms across multiple hereditary ataxias [7]. In particular, cerebellar microglia display a heightened immune-alert phenotype compared with microglia from other brain regions, raising the possibility that these cells actively shape regional vulnerability rather than merely responding to ongoing degeneration [7]. Consistent with this view, microglial activation has been documented in post-mortem tissue from several ataxia subtypes and in corresponding animal models [8,9,10,11]. In spinocerebellar ataxia type 1 (SCA1), early activation of microglia and astrocytes occurs within cerebellar circuits even when mutant protein expression is restricted to Purkinje cells, supporting a non-cell-autonomous contribution to disease progression [12]. Similar glial responses have been described in other spinocerebellar ataxias at later disease stages, suggesting that neuroinflammatory processes accompany degeneration across distinct genetic backgrounds [13]. A pronounced neuroimmune component has also been reported in ataxia–telangiectasia, where region-specific microglial dysfunction contributes to cerebellar susceptibility [14]. Recent single-cell analyses further suggest that microglial activation and inflammatory signaling precede Purkinje and granule cell death in this condition [15]. Neuroinflammatory signaling is also increasingly recognized in Friedreich’s ataxia (FRDA), the most common autosomal recessive ataxia of childhood. FRDA is primarily driven by mitochondrial dysfunction caused by frataxin deficiency; experimental and patient-derived studies indicate that the disease is also associated with oxidative stress, inflammatory signaling, reactive gliosis, and microglial dysfunction within vulnerable neuronal regions, particularly in the cerebellum, dentate nucleus, and spinal pathways. These observations suggest that neuroimmune alterations may amplify neuronal dysfunction downstream of metabolic stress through non-cell-autonomous mechanisms involving both microglia and astrocytes [10,14,16,17,18]. Together, these observations support the view that neuroinflammation acts as a disease-modifying component across genetically distinct ataxias, operating alongside cell-autonomous mechanisms to shape cerebellar circuit vulnerability. This perspective challenges the traditional notion of the central nervous system as strictly immune-privileged. Conversely, a growing body of literature suggests that the cerebellum undergoes active immune surveillance and exhibits region-specific features, including differences in blood–brain barrier properties, immune tolerance, and glial reactivity, that may enhance susceptibility to inflammatory insults [19]. The central hallmark of most forms of ataxias is the dysfunction or degeneration of Purkinje cells, a neuronal population characterized by increased sensitivity to cellular stressors due to high intrinsic firing rates, elaborate dendritic architecture, and elevated metabolic demands. In addition, accumulating evidence indicates that neuroinflammatory mechanisms contribute directly to Purkinje cell vulnerability [19,20], further supporting a mechanistic association between altered cerebellar microglial states and Purkinje cell dysfunction, with microglial activation driving Purkinje hyperexcitability and ataxic motor deficits in vivo [21]. Activated microglia and reactive astrocytes are consistently observed in affected cerebellar regions, where they influence neuronal survival and synaptic function through cytokine release, altered glia–neuron communication, and disruption of homeostatic support [12,13]. Notably, microglial activation often precedes overt Purkinje cell degeneration in hereditary ataxia models, suggesting the presence of an early phase of the disease during which circuit dysfunction may still be modifiable [7]. In this way, cerebellar neuroimmune alterations may have consequences beyond motor control by influencing cerebello-cortical network function, a circuit-level dimension increasingly implicated in cognitive and affective features of cerebellar disorders [22]. Nevertheless, experimental data caution against indiscriminate pharmacological approaches promoting immune suppression, since basal microglial signaling is required for synaptic refinement and circuit stability [7]. These findings support a shift from broad anti-inflammatory strategies toward calibrated modulation of neuroimmune tone. However, translating microglia-directed interventions into effective strategies remains challenging. Increasingly precise genetic and pharmacological approaches, including the targeting of disease-modifying microglial pathways such as TNF-α signaling, highlight the need for improved spatial and cellular specificity to minimize off-target effects, since TNF-α can directly modulate Purkinje cell excitability and cerebellar synaptic activity [22,23]. In addition, the therapeutic window represents a critical variable, particularly in hereditary ataxias, where neuroimmune alterations may emerge during pre-symptomatic stages. Early interventions should also consider the normal developmental roles of microglia, such as synaptic pruning and circuit refinement, because excessive or mistimed modulation may be ineffective or even aggravate developmental circuit abnormalities [24,25]. In this context, zebrafish (*Danio rerio*) are particularly useful for studying microglia in vivo. During embryonic and larval stages, optical transparency combined with transgenic reporter lines such as Tg(mpeg1:GFP), Tg(apoeb:GFP), and Tg(mfap4:tdTomato-CAAX) enables direct imaging of microglial morphology, migration, and phagocytic activity within the intact CNS [26,27,28]. These tools have been used to visualize microglia-mediated clearance of apoptotic neurons and to monitor glial responses during development, injury, and inflammation [27,29,30,31]. These features allow direct in vivo visualization of neuron–glia interactions and longitudinal assessment of microglial behavior within intact neural circuits. Zebrafish also provide robust behavioral readouts relevant to cerebellar function and motor coordination [32,33], together with opportunities to evaluate pharmacological and genetic modulation of neuroinflammatory pathways in a whole-organism context [26,27,28,29,30,31,34]. From an ethical perspective, zebrafish support the principles of the 3Rs (Replacement, Reduction, and Refinement), enabling early-stage mechanistic and pharmacological studies while potentially reducing reliance on higher-order mammalian models [35,36,37]. In particular, zebrafish embryos and larvae are generally considered to experience a lower degree of experimental burden and suffering compared with mammalian vertebrate models such as rodents, while still providing a vertebrate nervous system with conserved cellular and molecular pathways relevant to human disease. Their small size, external development, optical transparency, and suitability for high-throughput approaches further contribute to reducing animal numbers and invasive procedures in biomedical research [35,36,37]. By integrating mechanistic insights into cerebellar neuroinflammation with experimentally tractable zebrafish models, this review aims to provide a conceptual and methodological framework for investigating hereditary ataxias. In this context, flavonoids are considered a biologically coherent class of compounds with the potential to modulate microglial reactivity, pro-inflammatory signaling, and oxidative stress pathways, all of which are increasingly implicated in cerebellar dysfunction across hereditary ataxias [38,39,40,41]. In inflammatory contexts, microglial activation can engage NF-κB-dependent transcriptional programs, promoting the release of cytokines such as TNF-α and IL-1β that may alter neuronal excitability, synaptic transmission, and oxidative stress responses [42]. Naringenin has been shown to target several inflammatory signals involved in the neuroinflammatory responses, including mitogen-activated protein kinase (MAPK), suppressor of cytokine signaling 3 (SOCS-3), NF-κB and signal transducer and activator of transcription-1 (STAT-1) [43,44,45,46], supporting its use as a tool to probe neuroimmune modulation rather than broad immune suppression. Naringenin has been examined across multiple vertebrate systems, including zebrafish, where it modulates NF-κB-associated signaling and stress-responsive pathways [47,48]. The availability of in vivo data and its relatively defined molecular profile support its use as an experimental probe to investigate neuroimmune balance rather than as a speculative therapeutic endpoint. In the context of this review, the term “early” refers primarily to pre-degenerative and early pathogenic neuroimmune alterations emerging before overt neuronal loss, rather than exclusively to developmental or pediatric disease onset. This review is based on three interconnected concepts. First, neuroimmune alterations in hereditary cerebellar ataxias may emerge before overt neuronal degeneration and actively contribute to cerebellar circuit dysfunction rather than representing purely secondary phenomena. Second, the region-specific properties of cerebellar microglia may influence the selective vulnerability of Purkinje cell circuits across genetically distinct ataxias. Third, zebrafish models provide a uniquely tractable in vivo platform to investigate early neuroimmune dynamics and to experimentally test modulatory strategies targeting inflammatory tone during pre-degenerative stages. Within this framework, flavonoids such as naringenin are discussed primarily as mechanistic probes of neuroimmune modulation rather than as direct therapeutic endpoints. The literature discussed in this narrative review was identified through searches in PubMed, Scopus, and Web of Science using combinations of terms related to hereditary cerebellar ataxias, neuroinflammation, microglia, zebrafish, flavonoids, and naringenin. Additional studies were identified through reference screening of relevant articles. Priority was given to original experimental studies, recent reviews, transcriptomic analyses, and in vivo investigations published primarily between 2010 and 2026, while earlier foundational studies were included when considered conceptually or methodologically relevant to cerebellar neuroimmune biology and zebrafish disease modeling.

## 2. The Cerebellum and Neuroinflammation

The cerebellum is a highly organized brain structure that contains the majority of neurons in the human brain and is characterized by a conserved cytoarchitecture and a precise spatial arrangement of neuronal and glial populations [49]. Although it has traditionally been associated with motor coordination and sensorimotor integration, the cerebellum is now recognized as a key hub within distributed networks supporting cognitive and affective functions, enabled by extensive reciprocal connections with cortical and subcortical regions [50]. This functional diversity is sustained by a complex cellular environment in which neurons and glial cells continuously interact to preserve circuit stability while allowing adaptive plasticity. Immune-related processes also play important roles in cerebellar development, synaptic maturation, and the maintenance of tissue homeostasis under physiological conditions [51]. Among resident glial populations, microglia and astrocytes actively contribute to cerebellar circuit formation and function [52]. Cerebellar microglia exhibit distinct regional distributions and phenotypic profiles compared with those described in other brain areas, reflecting the unique cytoarchitecture and connectivity of cerebellar circuits [19,53,54]. This regional specialization suggests that their functions are adapted to local circuit demands and include synaptic maintenance, regulation of neuronal excitability, and immune surveillance under physiological conditions. Importantly, regional heterogeneity is increasingly recognized as a general feature of microglial populations throughout the CNS rather than an exclusive property of cerebellar microglia. Within this broader framework, cerebellar microglia appear to constitute a particularly specialized population characterized by distinct morphological, transcriptional, and functional properties compared with cortical microglia. In vivo imaging studies demonstrated that cerebellar microglia exhibit reduced ramification, increased motility, and enhanced surveillance dynamics, particularly in relation to Purkinje neurons [53]. Furthermore, alterations in microglial density, morphology, and process complexity are increasingly recognized as informative indicators of cerebellar microglial states during neurodevelopment, neurodegeneration, and inflammatory responses [20,55]. Recent transcriptomic and functional analyses also identified cerebellar-specific immune and metabolic signatures associated with enhanced immune responsiveness and neuroregulatory functions [20]. Furthermore, emerging evidence suggests that microglial heterogeneity within the cerebellum itself may also reflect compartment-specific differences between white matter- and gray matter-associated microglia, which are known to display distinct molecular and functional properties. Together, these observations support the concept that cerebellar neuroimmune specialization may contribute to the selective vulnerability of cerebellar circuits observed in hereditary ataxias. Recent genome-wide analyses have shown that microglia progress through distinct developmental phases, from early embryonic stages to a mature adult state, each regulated by specific transcriptional programs that shape their functional properties [51]. These findings indicate that microglia remain functionally responsive across developmental stages, with roles that extend beyond classical immune surveillance and also affect neuronal function under both physiological and pathological conditions [51]. At the molecular level, cerebellar microglia display features that distinguish them from those found in other brain regions. Transcriptomic analyses in adult mice revealed that these cells show a region-specific molecular signature enriched for genes involved in immune surveillance and antigen processing, including pattern-recognition receptors (e.g., Clec7a, Clec4e), interferon-related genes (Stat1, Irf7) and MHC class I/II components, suggesting a constitutively vigilant state rather than a classical injury-induced activation [54]. This immune-alert profile is accompanied by elevated expression of metabolic and mitochondrial regulators and antioxidant enzymes, indicating an enhanced energetic and redox capacity in the cerebellar niche. Complementary functional and epigenetic analyses further demonstrate that cerebellar microglia exhibit increased lysosomal and clearance activity, with enrichment of genes linked to phagocytosis and lipid metabolism, supporting the concept of a regionally specialized, homeostatically primed phagocytic phenotype [56]. Notably, this immune-oriented profile appears to intensify with age, as cerebellar microglia display a stronger age-related increase in immune gene expression compared with cortical or striatal microglia. Such long-term immune priming may represent a factor contributing to the late emergence of cerebellar ataxias, especially in forms where microglial alterations precede clinical symptoms [12]. Clarifying the mechanisms that shape these region-specific characteristics will be important for understanding how microglia influence cerebellar pathology. Astrocytes represent another essential component of the cerebellar neuroimmune environment. In particular, Bergmann glia, an astrocytic population unique to the cerebellar cortex, form a close structural and functional association with Purkinje cells [57]. Through regulation of extracellular ion homeostasis, neurotransmitter clearance, and metabolic support, Bergmann glia play a central role in shaping Purkinje cell excitability and synaptic integration. These astrocytes are also immune-responsive cells that can produce cytokines and modulate neuroinflammatory signaling in response to changes in the local microenvironment [57]. Although protected by the blood–brain barrier, cerebellar vasculature and perivascular compartments provide interfaces for molecular exchange and immune communication between the central nervous system and the periphery, potentially contributing to region-specific sensitivity to inflammatory signals [19,53]. Together, these features suggest that cerebellar circuits may be particularly sensitive to systemic inflammatory signals, even in the absence of overt barrier disruption [19]. Overall, these observations indicate that the cerebellum hosts a functionally engaged and regionally specialized neuroimmune system rather than a uniform or passive immune environment. This persistent immune-alert state in adulthood may therefore contribute to the increased vulnerability of cerebellar circuits in ataxic disorders.

## 3. Purkinje Cells as a Vulnerable Node in the Cerebellar Neuroimmune Environment

**Figure 1:**
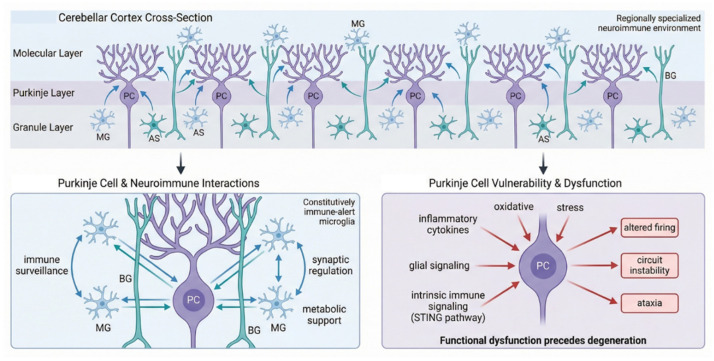
Cerebellar Neuroimmune Specialization and Early Purkinje Cell Dysfunction. The (**upper panel**) illustrates the layered organization of the cerebellar cortex and the regionally specialized neuroimmune environment involving Purkinje cells, microglia (MG), and Bergmann glia (BG). The (**lower left panel**) summarizes homeostatic neuroimmune interactions that contribute to immune surveillance, synaptic regulation, ion homeostasis, metabolic support, and context-dependent microglial states within cerebellar circuits. The (**lower right panel**) illustrates mechanisms potentially contributing to early Purkinje cell dysfunction, including inflammatory cytokines, oxidative stress, mitochondrial dysfunction, DAMP (Damage-Associated Molecular Patterns) release, glial signaling, and intrinsic STING-associated immune signaling. These neuroimmune alterations may promote altered neuronal firing, circuit instability, and cerebellar motor dysfunction before overt neurodegeneration. The figure also highlights the concept that cerebellar microglial states operate along a dynamic functional spectrum ranging from homeostatic to maladaptive neuroimmune conditions. This figure was generated with the assistance of FigureLabs AI Agent (https://www.figurelabs.ai/, accessed on 25 May 2026) for Scientific Illustration and subsequently curated by the authors.

Within the regionally specialized neuroimmune environment of the cerebellum, not all neuronal populations are equally affected. Among the cellular components of the cerebellar cortex, Purkinje cells represent a particularly vulnerable neuronal population. As the sole output neurons of the cerebellar cortex, Purkinje cells provide inhibitory projections to the deep cerebellar and vestibular nuclei, which constitute the main output structures of the cerebellum [58]. The distinctive physiological properties that enable Purkinje cells to perform this integrative role, including high intrinsic firing rates, extensive dendritic arborization, and sustained synaptic activity, also impose exceptional metabolic and regulatory demands. Accordingly, selective vulnerability of Purkinje cells is a consistent feature across a wide range of cerebellar disorders, in which early functional impairment often precedes progressive degeneration [59]. This susceptibility is unlikely to be explained entirely by cell-autonomous mechanisms, as Purkinje cell degeneration frequently occurs in conditions in which disease-associated mutations are broadly expressed or primarily affect other cell types. These observations suggest that non-cell-autonomous factors contribute critically to shaping Purkinje cell vulnerability. Purkinje cells are embedded within a dense and highly specialized glial network that exerts a profound influence on their function and survival [60]. Bergmann glial cells form an intimate structural and functional association with Purkinje cell dendrites, regulating metabolic support and extracellular ion homeostasis, thereby stabilizing excitability and preserving circuit integrity under physiological conditions [61]. Microglia constitute another key component of the Purkinje cell microenvironment. By dynamically surveying cerebellar circuits and responding to fluctuations in neuronal activity and tissue homeostasis, microglia modulate synaptic function and neuronal excitability, exerting direct neuromodulatory effects that extend beyond their classical immune role [61]. Rather than acting as uniformly detrimental effectors, cerebellar microglia operate along a context-dependent functional spectrum, balancing neuroregulatory and neuroinflammatory states [20]. Given the strict reliance of cerebellar output on the temporal precision of Purkinje cell firing, even subtle immune-mediated perturbations can result in pronounced alterations of circuit function. Importantly, inflammatory modulation of Purkinje cell activity does not necessarily translate into overt ataxia. Experimental studies in mammalian models indicate that acute microglial activation can transiently enhance Purkinje cell firing and synaptic activity, whereas chronic developmental inflammatory stress may reduce Purkinje cell excitability without immediately inducing motor ataxia phenotypes [22,62]. These observations suggest that neuroimmune signaling may initially alter cerebellar circuit function in subtle and potentially reversible ways before overt neurodegeneration and clinical ataxia emerge. Moreover, the high metabolic demand and sustained electrical activity of Purkinje cells render them particularly susceptible to oxidative stress and excitotoxicity, lowering the threshold at which inflammatory challenges translate into functional impairment. Functional disturbances in Purkinje cells often arise before overt neuronal loss, supporting the notion that Purkinje cell dysfunction represents an early and potentially reversible stage of pathology. Potential triggers of these early neuroimmune alterations likely include intracellular protein aggregation, mitochondrial dysfunction, oxidative stress, defective DNA repair pathways, altered neuronal activity, and the release of damage-associated molecular patterns from stressed neurons and glial cells [63,64]. Recent studies have expanded this framework by demonstrating that Purkinje cells are not merely passive targets of inflammation but can autonomously activate intrinsic immune signaling pathways. Expression of the innate immune adaptor STING (TMEM173), classically associated with cytosolic DNA sensing in immune cells, has been directly identified in Purkinje cells [65]. Selective activation of STING signaling in Purkinje cells is sufficient to induce progressive cerebellar degeneration and ataxia, independently of microglial activation or type I interferon signaling. Importantly, early disruption of autonomous firing and pacemaking properties precedes overt structural degeneration, further supporting the idea that functional circuit alterations may emerge before irreversible neuronal loss [65]. Together, these findings support a model in which Purkinje cells’ vulnerability arises from their position at the interface between cerebellar circuit function and neuroimmune regulation, highlighting the potential relevance of strategies aimed at modulating, rather than suppressing, neuroimmune mechanisms to preserve cerebellar homeostasis in disease. Emerging evidence indicates that microglial functional states are dynamically shaped by transcriptional, metabolic, and chromatin-associated mechanisms, enabling context-dependent inflammatory plasticity and state transitions, as reviewed in [66,67]. Experimental studies in neurodegenerative and neuroinflammatory conditions further suggest that modulation of maladaptive microglial transcriptional programs may influence disease-associated neuroimmune phenotypes and inflammatory persistence [68]. Although these concepts remain largely unexplored in hereditary cerebellar ataxias, the distinctive immune-alert properties of cerebellar microglia raise the possibility that reshaping glial functional states could influence Purkinje cell vulnerability and cerebellar circuit stability. These interconnected neuroimmune mechanisms and their proposed contributions to cerebellar vulnerability are summarized in Figure 1.

## 4. Modeling Neuroimmune Mechanisms in the Cerebellum: The Zebrafish Advantage

The growing recognition that neuroinflammation contributes to cerebellar dysfunction through context-dependent and potentially reversible mechanisms has important implications for therapeutic research. Rather than broadly suppressing immune activity, a more useful strategy may be to modulate chronic inflammatory signaling while preserving essential microglial functions. Evidence from mammalian models further highlights the complexity of therapeutically targeting microglial pathways. In SCA1 mouse models, both pharmacological microglial depletion and genetic attenuation of NF-κB signaling reduce inflammatory markers, such as TNF-α, but fail to significantly rescue motor deficits or cerebellar neuropathology [69]. Moreover, excessive attenuation of immune-related signaling pathways may also interfere with physiological processes involved in cerebellar circuit maintenance, including synaptic refinement and neuron–glia communication [69]. Since the LysM-Cre model used in this study is not strictly microglia-specific, these findings should be interpreted more broadly within the context of neuroimmune and myeloid-associated signaling pathways rather than selective microglial suppression. Overall, these observations support the idea that disruption of neuroimmune homeostasis may destabilize cerebellar circuitry. Consequently, therapeutic strategies must account for spatial specificity, cellular targeting, and the temporal window of intervention, particularly during pre-symptomatic stages in hereditary ataxias. This requires experimental models that can capture neuroimmune interactions dynamically, detect early functional changes before overt neurodegeneration, and support systematic in vivo testing of candidate modulatory compounds. While mammalian models have been instrumental in elucidating disease mechanisms, they present inherent limitations in terms of scalability, experimental throughput, and feasibility for pharmacological screening. In this setting, zebrafish (*Danio rerio*) provide a valuable complementary model, combining vertebrate brain organization with optical accessibility, genetic tractability, and suitability for high-throughput in vivo screening [70,71,72]. In addition, the ethical imperative to adhere to the principles of Replacement, Reduction, and Refinement (the 3Rs) further supports the use of zebrafish in neuroimmune and translational research [35]. Despite differences in overall brain size and complexity, the cerebellum exhibits a high degree of structural and functional conservation across vertebrate species. Core organizational features are preserved from teleosts to mammals, including a layered cerebellar cortex, defined neuronal subtypes, and conserved circuit motifs [73]. In zebrafish, the cerebellum develops early and displays key anatomical hallmarks of vertebrate cerebellar organization, supporting its use as a translational model for studying cerebellar function and dysfunction [74]. The zebrafish cerebellum has three lobes: the corpus cerebelli (CCe), the valvula cerebelli (Va), and the vestibulolateral lobe. CCe and Va have tri-lamellar structures comprising the granule cell, Purkinje cell, and molecular layers. These layers have the same orientation in CCe as in the mammalian cerebellum [75]. Representative schematic comparisons between zebrafish and mammalian cerebellar organization, including Purkinje-cell circuitry and transgenic approaches for modeling spinocerebellar ataxias, have been comprehensively illustrated in previous zebrafish cerebellum studies and dedicated methodological reviews [76]. Importantly, despite species-specific differences in cerebellar complexity and foliation, zebrafish preserve key cellular and functional features of vertebrate cerebellar circuits, including Purkinje-like neurons, glutamatergic granule cells, climbing fiber organization, and conserved neuron–glia interactions [76]. These conserved characteristics, together with optical accessibility and genetic tractability, support the translational relevance of zebrafish models for investigating early cerebellar dysfunction and neuroimmune dynamics in vivo. In particular, Purkinje-like neurons in zebrafish share essential morphological, molecular, and functional properties with their mammalian counterparts. These neurons exhibit elaborate dendritic arborization, express conserved transcriptional markers, and occupy analogous positions within cerebellar circuits, where they integrate synaptic inputs and regulate downstream cerebellar output [77]. Functional studies indicate that zebrafish Purkinje-like neurons display intrinsic firing activity and synaptic properties comparable to those described in mammals, enabling investigation of early functional alterations of cerebellar circuits, and are supported by radial glial cells that share developmental and functional features with mammalian Bergmann glia [78]. Importantly, radial glia and microglia in zebrafish respond dynamically to inflammatory stimuli and neuronal stress, making them suitable for studying how glial responses may influence cerebellar circuits [78,79,80]. This conserved glial architecture enables in vivo investigation of neuroimmune interactions within a cerebellar context that closely parallels mammalian systems. Finally, the development of a functional BBB in zebrafish offers additional experimental opportunities [81], providing a valuable platform for assessing central nervous system bioavailability and pharmacokinetics in vivo. The temporal accessibility of the developing zebrafish BBB enables evaluation of candidate compounds across different stages of barrier maturation, facilitating the evaluation of molecules with central nervous system access that may later be tested for effects on cerebellar neuroimmune processes [81]. Overall, these features support the use of zebrafish not as a substitute for mammalian therapeutic validation, but as an experimentally tractable system for defining functional windows of neuroimmune modulation and identifying conditions in which circuit stability can be preserved before irreversible degeneration develops. Nevertheless, some important limitations should also be considered when modeling hereditary cerebellar ataxias in zebrafish. Although zebrafish reproduce several conserved aspects of cerebellar organization, neuronal vulnerability, locomotor dysfunction, and glial responses, they cannot fully recapitulate the anatomical complexity and long-term progressive course of human cerebellar degeneration. Certain clinical manifestations frequently observed in patients, including higher-order cognitive impairment, complex speech alterations, and multisystemic features associated with peripheral neuropathy or extracerebellar degeneration, remain difficult to model in larval zebrafish paradigms. In addition, differences in lifespan, cerebellar architecture, and adaptive immune system maturation may influence the translational interpretation of neuroimmune mechanisms. Therefore, zebrafish models should primarily be viewed as complementary systems for mechanistic dissection and early-stage therapeutic prioritization rather than as complete replicas of human hereditary ataxias.

## 5. Transgenic and Imaging Tools for In Vivo Analysis of Cerebellar Neuroimmune Dynamics in Zebrafish

A distinctive strength of the zebrafish model lies not only in its suitability for high-throughput in vivo screening, but also in the wide availability of genetically modified lines that enable direct visualization and manipulation of defined neuronal and glial populations within intact cerebellar circuits. This genetic accessibility makes it possible to investigate neuroimmune dynamics in real time, capturing cellular behaviors that are still difficult to observe in other vertebrate systems. Transgenic zebrafish lines expressing fluorescent reporters in Purkinje-like neurons [73,76,82], radial glia [83], and microglia [26,27,28,84] allow continuous in vivo monitoring of cerebellar organization and neuron–glial interactions under both physiological and inflammatory conditions (summarized in Table 1). In particular, microglial reporter lines make it possible to directly observe cellular morphology, motility, and spatial interactions with cerebellar neurons, revealing rapid and dynamic responses to changes in neuronal activity, tissue stress, or inflammatory stimuli. These observations help distinguish transient adaptive responses from those leading to circuit dysfunction. Most importantly, these readouts capture early functional and cellular alterations that precede overt neurodegeneration, offering a sensitive window into disease-relevant processes. In addition, transgenic strategies can be used to generate cell-type-specific expression systems, enabling selective modulation of neuronal or glial gene expression to clarify unresolved links between neuroimmune interactions and cerebellar function. When combined with behavioral assays and high-throughput screening paradigms, these approaches offer a robust framework to systematically investigate neuroimmune mechanisms and prioritize candidate modulators in a vertebrate context.

## 6. Modeling Hereditary Cerebellar Ataxias in Zebrafish: Genetic Strategies and Functional Readouts

Building on the experimental tools described above, zebrafish models of cerebellar dysfunction provide a tractable disease context in which functional impairment, neuronal vulnerability, and non-cell-autonomous mechanisms can be investigated in vivo. Over the past decade, a growing number of zebrafish models have been developed to study hereditary ataxias [32,33] and related cerebellar disorders [89], capturing specific yet disease-relevant features such as impaired motor coordination, altered cerebellar circuit activity, and selective vulnerability of Purkinje-like neurons. Importantly, many of these models exhibit early functional deficits in the absence of widespread neuronal loss, allowing the investigation of dynamic cellular processes that may precede irreversible degeneration. Recent comprehensive analyses have highlighted the diversity of zebrafish approaches used to model cerebellar ataxias, including stable transgenic lines, CRISPR/Cas9-generated knockouts, F0 crispants, and targeted expression of disease-associated mutant proteins in Purkinje-like neurons [89]. Rather than reproducing the full clinical spectrum of human ataxias, these models are particularly informative for dissecting conserved mechanisms of cerebellar vulnerability, circuit dysfunction, and adaptive or maladaptive responses within the cerebellar microenvironment. Here, we focus on zebrafish models in which cerebellar dysfunction and/or ataxia-like motor phenotypes were explicitly assessed, providing experimentally actionable systems for downstream mechanistic and therapeutic studies (summarized in Table 2). Models primarily limited to developmental expression analyses, transient morpholino phenotypes, or studies lacking direct assessment of cerebellar dysfunction and/or motor coordination deficits are not included in Table 2. Accordingly, additional zebrafish models involving genes associated with SCA2 (atxn2) [90], SCA7 (atxn7) [91], or CACNA1A-related disorders [92,93] were not included when direct cerebellar dysfunction or ataxia-related functional phenotypes had not yet been formally characterized in vivo. Importantly, although neuroimmune or glia-related mechanisms have not yet been systematically assessed in several of these models, independent evidence from cellular and mammalian systems supports a contribution of neuroinflammatory processes to the corresponding disease contexts. In this light, zebrafish models should be viewed as still underutilized experimental platforms, in which the integration of disease-relevant genetic backgrounds with transgenic neuroimmune reporter lines and circuit-level functional readouts represents a powerful strategy to rapidly investigate glia-related mechanisms and prioritize candidate compounds for subsequent validation in mammalian and human-derived in vitro models. Despite the experimental tractability of currently available zebrafish hereditary ataxia models, the integration of these genetic backgrounds with microglial and macrophage transgenic reporter lines, including Tg(mpeg1:GFP) and Tg(mfap4:tdTomato-CAAX), remains largely unexplored. As a result, formal in vivo characterization of cerebellar neuroimmune dynamics in hereditary ataxia zebrafish models is still missing, representing both a major limitation in the current literature and a significant experimental opportunity for future studies.

## 7. Neuroimmune Modulation in Hereditary Ataxias: A Proposed Zebrafish Experimental Framework

**Figure 2:**
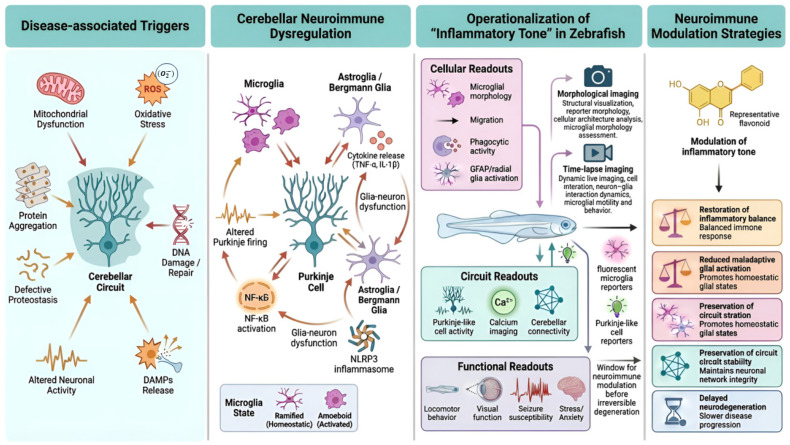
Conceptual framework for neuroimmune modulation in hereditary cerebellar ataxias using zebrafish models. The figure summarizes the proposed relationship between disease-associated stressors, cerebellar neuroimmune dysregulation, and zebrafish-based operationalization of inflammatory tone in hereditary cerebellar ataxias. Mitochondrial dysfunction, oxidative stress, altered neuronal activity, defective proteostasis, DNA damage, and DAMP release may contribute to maladaptive neuroimmune signaling involving microglia, Bergmann glia, inflammatory cytokines, NF-κB signaling, and inflammasome-associated pathways, ultimately affecting Purkinje cell function and cerebellar circuit stability. The central panel illustrates representative zebrafish cellular, circuit, and functional readouts that can be used to investigate neuroimmune states in vivo, including live imaging approaches, calcium imaging, locomotor behavior, visual function, seizure susceptibility, and stress-related phenotypes. The right panel summarizes the rationale for neuroimmune modulation strategies aimed at preserving circuit stability and delaying neurodegeneration during pre-degenerative stages. This figure was generated with the assistance of FigureLabs AI Agent (https://www.figurelabs.ai/, accessed on 25 May 2026) for Scientific Illustration and subsequently curated by the authors.

The evidence discussed in the preceding sections converges on a central concept: cerebellar dysfunction in hereditary ataxias arises from the interplay between intrinsic neuronal vulnerability and extrinsic modulatory processes within the local microenvironment. While genetic defects define disease onset, increasing evidence from cellular and mammalian models indicates that neuroinflammatory and glia-related mechanisms can shape disease features, influence circuit stability, and modulate the tempo of functional decline. This perspective shifts the therapeutic focus away from irreversible neuronal loss toward strategies that target dynamic and potentially reversible disease processes. Despite this conceptual advance, neuroinflammation has been only marginally explored as a modifiable target in zebrafish models of cerebellar disorders. As highlighted above, many genetically defined zebrafish models faithfully recapitulate cerebellar dysfunction and ataxia-like phenotypes, yet immune-related mechanisms have rarely been incorporated as experimental endpoints. This gap does not reflect technical limitations, but rather a historical emphasis on neuron-intrinsic pathways. The availability of transgenic neuroimmune reporters, live imaging approaches, and scalable behavioral assays makes zebrafish an ideal system to directly test whether modulation of glia-related mechanisms can influence disease features in vivo. In this context, the challenge is not to indiscriminately suppress inflammatory signaling, but to identify compound classes capable of fine-tuning neuroimmune responses while preserving essential homeostatic functions. Such an approach is particularly relevant in the cerebellum, where tightly regulated neuron–glia interactions are required for circuit precision and adaptive plasticity. Selecting biologically coherent and experimentally tractable modulators, therefore, represents a critical step toward translating mechanistic insights into testable strategies. Among the wide range of compounds with reported anti-inflammatory activity, flavonoids constitute a large and heterogeneous class of naturally occurring polyphenols [101]. Numerous flavonoids, including quercetin [102], kaempferol [103], and taxifolin [104], have been investigated across diverse experimental systems for their anti-inflammatory, antioxidant, and neuroprotective properties. This body of literature underscores the biological relevance of flavonoids [101,104], but also highlights the challenge of providing a comprehensive overview of such a chemically and functionally diverse group within a single review. Several studies indicate that flavonoids directly regulate microglial activation states, cytokine production, and inflammatory signaling cascades, supporting the idea that microglia represent a primary cellular target of their neuroprotective actions [39,40,105,106]. Importantly, these compounds do not act as broad immunosuppressive agents but instead fine-tune microglial responses to inflammatory and metabolic stressors, thereby preserving neuronal function and limiting secondary tissue damage. Mechanistically, flavonoids modulate key signaling pathways governing microglial activation, including NF-κB, MAPK, and Nrf2, resulting in attenuation of pro-inflammatory gene expression alongside induction of antioxidant and cytoprotective programs [107,108,109]. These pathways are increasingly recognized as convergent regulators of oxidative stress, inflammatory amplification, and microglia-mediated neuronal vulnerability across neurodegenerative conditions, as extensively discussed in previous comparative reviews addressing flavonoid-mediated neuroimmune modulation [41,110,111]. This mode of action is particularly relevant in neurodegenerative disorders, including cerebellar ataxia, where excessive or prolonged glial activation may amplify neuronal vulnerability without representing the primary etiological trigger. Although the flavonoid family comprises a wide and chemically diverse group of compounds extensively studied in neurodegenerative contexts, this review does not aim to provide a comprehensive survey, but rather uses flavonoids as a conceptual and mechanistic framework to discuss neuroimmune modulation [112]. Given the availability of dedicated comparative reviews discussing flavonoid subclasses, neuroprotective properties, and partially overlapping anti-neuroinflammatory mechanisms, the present discussion does not aim to provide a broad comparative overview of flavonoid pharmacology. Instead, it focuses on naringenin as a representative flavanone selected for its experimental tractability, relatively well-characterized neuroimmune mechanisms, and the availability of convergent evidence across cellular systems, mammalian studies, zebrafish models, and genetically driven motor neurodegenerative disorders, including hereditary spastic paraplegia models, where naringenin has been shown to modulate ER stress responses, organelle homeostasis, and locomotor dysfunction [113,114]. Importantly, naringenin has been shown to modulate microglial responses in a context-dependent manner while preserving key homeostatic functions [115]. These features support its use as a suitable compound to investigate neuroimmune dynamics in an integrated in vivo setting. The conceptual framework discussed in this section, integrating disease-associated triggers, cerebellar neuroimmune dysregulation, zebrafish-based functional operationalization, and neuroimmune modulation strategies, is summarized in Figure 2.

### Naringenin as a Mechanistic Probe for Neuroimmune Modulation

Within the proposed zebrafish experimental framework discussed throughout this review, neuroimmune-modulating compounds may provide useful tools to investigate how inflammatory signaling influences cerebellar vulnerability and circuit dysfunction during pre-degenerative stages. In this context, naringenin is discussed primarily as a mechanistically informative and hypothesis-generating flavonoid rather than as a validated therapeutic strategy for hereditary cerebellar ataxias. Building on this rationale, a growing body of experimental evidence supports a role for naringenin as a modulator of neuroinflammatory and neurodegenerative processes [116,117]. Naringenin exhibits several beneficial effects on human health, including the reduction in lipid peroxidation and protein carbonylation, the enhancement of carbohydrate metabolism, and the strengthening of antioxidant defenses through the scavenging of reactive oxygen species [118]. Beyond these well-established antioxidant properties, naringenin exerts direct effects on immune signaling pathways that are highly relevant to central nervous system pathology [43,46,119]. In cellular models, naringenin attenuates microglial activation and pro-inflammatory cytokine release by modulating NF-κB, MAPK, and JAK/STAT signaling pathways, while promoting a shift toward anti-inflammatory or homeostatic microglial phenotypes [43,119]. Additional studies further indicate that naringenin-associated neuroprotective effects involve attenuation of oxidative stress amplification and inflammatory mediators linked to microglia-driven neuronal toxicity, supporting coordinated regulation of interconnected neuroimmune and stress-responsive pathways rather than isolated modulation of single signaling cascades [43,46,119,120]. These effects have been consistently observed in lipopolysaccharide-stimulated microglial cultures and neuron–microglia co-culture systems, where naringenin reduces the production of TNF-α, IL-1β, and reactive oxygen species while preserving neuronal viability [46]. In vivo studies further support the neuroprotective potential of naringenin in models of neurodegeneration. In rodent models of Alzheimer’s disease, naringenin administration ameliorates cognitive impairment, reduces oxidative stress and neuroinflammatory markers, and modulates synaptic and apoptotic pathways associated with disease progression [121]. Similar neuroprotective effects have been reported in models of epilepsy, ischemic injury, spinal cord injury, and age-associated cognitive decline, indicating that naringenin targets convergent inflammatory and stress-related mechanisms shared across neurodegenerative conditions [122,123,124]. Importantly, several of these studies explicitly implicate microglial regulation as a key component of naringenin’s mechanism of action, positioning this flavanone as a mediator integrating immune modulation and neuronal survival. From a translational perspective, naringenin is distinguished from many other flavonoids by its favorable safety profile and its evaluation in human studies [118]. Evidence supporting the in vivo tractability of naringenin is also provided by studies performed in zebrafish models (Table 3). Although these investigations were not specifically designed to address cerebellar circuits or ataxia-related phenotypes, they collectively demonstrate that naringenin can be effectively administered in a vertebrate organism amenable to genetic manipulation, live imaging, and behavioral analyses. One of the most comprehensive recent studies showed that pre-treatment with naringenin significantly protected zebrafish larvae against β-N-methylamino-L-alanine (BMAA)-induced neuromuscular toxicity, preserving muscle architecture and neuromuscular junction integrity. This protective effect was accompanied by normalization of myogenic gene expression and suppression of inflammatory mediators, including NF-κb and il-1β, supporting a combined anti-inflammatory and tissue-protective mechanism in vivo [47]. Behavioral and neurotoxicity models further support the in vivo relevance of naringenin. In adult zebrafish subjected to acute stress exposure, naringenin treatment significantly reduced anxiety-like behavior, as assessed by increased exploration in the upper zone of the novel tank test and greater time spent in the light compartment of the light–dark preference test [125]. Similarly, in a bisphenol A (BPA)-induced neurotoxicity model, co-supplementation with naringenin ameliorated anxiety-like and neurobehavioral alterations while modulating oxidative stress parameters, suggesting a stress-buffering and neuroprotective effect [48]. In larval seizure models induced by pentylenetetrazole (PTZ), pretreatment with naringenin reduced hyperactive locomotor responses and modulated molecular markers associated with oxidative stress and neuronal activation [123]. Although microglial activation was not directly assessed, the modulation of stress- and inflammation-related pathways supports a role for naringenin in excitotoxic and neuroinflammatory contexts. Naringenin has also demonstrated neuroprotective properties in a zebrafish model of dopaminergic neurodegeneration induced by 6-hydroxydopamine (6-OHDA), where treatment improved locomotor performance and attenuated oxidative stress-related alterations [126]. These findings indicate that naringenin can influence conserved molecular pathways involved in neurodegenerative processes. Beyond strictly neuronal paradigms, naringenin was reported to exert systemic and metabolic effects in zebrafish. In larval models of ethanol-induced hepatic injury, the compound reduced steatosis, apoptosis, and dysregulation of lipid metabolism [127]. In a hyperglycemia-induced model of early diabetic retinopathy, naringenin decreased oxidative stress markers and vascular endothelial growth factor (VEGF) expression, further supporting modulation of redox-sensitive and inflammatory pathways in vivo [128]. Overall, these findings converge on the idea that naringenin acts on conserved molecular stress-response and inflammatory pathways across multiple biological contexts in zebrafish. Importantly, although evidence in hereditary cerebellar ataxias is still emerging, naringenin has already shown neuroprotective and anti-inflammatory effects in other genetically driven motor neurodegenerative disorders, including hereditary spastic paraplegia models [113,114], supporting its potential relevance as a neuroimmune-modulating strategy for hereditary cerebellar ataxias.

## 8. Discussion

A central theme emerging from the literature is that cerebellar dysfunction in hereditary ataxias reflects a dynamic interplay between vulnerable Purkinje cells and their surrounding glial microenvironment rather than solely cell-autonomous neuronal degeneration [21]. Microglial activation, astrocytic reactivity, redox imbalance, and stress-responsive signaling cascades have been implicated across multiple genetic ataxias [7], supporting the idea that neuroimmune mechanisms actively contribute to disease progression rather than merely accompanying it. Region-specific transcriptomic analyses indicate that cerebellar microglia display a constitutively “hyper-vigilant” immune phenotype compared with microglia from other brain regions, characterized by enhanced immune-alert and metabolic gene expression [7]. Rather than representing a classical injury-driven activation state, this profile suggests an intrinsically lower threshold for immune responsiveness within cerebellar circuits. This concept is consistent with the idea that the cerebellum represents a region with specialized immune regulatory properties and distinct mechanisms of immune tolerance [19]. Consistent with this idea, microglial activation precedes overt Purkinje cell degeneration in several ataxia models, including SCA1, SCA21, and SCA6 [8,9,69], as well as in the ATM knockout rat model of ataxia–telangiectasia [11]. This temporal dissociation reinforces the notion that inflammatory signaling is involved in early circuit dysfunction, not only in downstream degenerative stages. At the same time, experimental evidence cautions against interpreting microglial activation as uniformly detrimental. Genetic attenuation of NF-κB signaling in microglia can itself induce ataxia-like deficits in the absence of Purkinje cell degeneration [7], consistent with studies showing that microglia regulate climbing fiber pruning and inhibitory synaptic maturation during cerebellar development [129]. Together, these observations highlight the importance of balanced neuroimmune regulation for maintaining cerebellar circuit stability. Accordingly, modulation of inflammatory tone, rather than complete inhibition, may represent a more biologically coherent strategy. An important unresolved challenge is defining which neuroimmune states remain adaptive and which become maladaptive during the transition from early circuit dysfunction to overt neurodegeneration. In this context, zebrafish models offer a unique opportunity to combine hereditary ataxia genetic backgrounds with real-time neuroimmune reporters, longitudinal imaging approaches, and functional behavioral profiling within experimentally accessible developmental windows. Beyond descriptive inflammatory signatures, these approaches may help identify mechanistic thresholds linking microglial state transitions to Purkinje-like cell dysfunction and cerebellar circuit instability. Such experimental paradigms could also facilitate temporally controlled pharmacological interventions aimed at modulating inflammatory tone without disrupting physiological neuroimmune functions. While mammalian models have been essential for defining inflammatory cascades, they provide limited temporal resolution for capturing early functional disturbances preceding degeneration. Zebrafish models offer a complementary approach by enabling longitudinal analysis of neuron–glia interactions and microglial behavior during pre-degenerative stages [30,31,130]. Although several zebrafish models reproduce early cerebellar dysfunction before widespread neuronal loss [72,85], neuroimmune mechanisms remain comparatively underexplored, as most studies have focused primarily on neuronal phenotypes without systematically addressing glial activation or inflammatory signaling. From this perspective, the central question shifts from the presence of inflammation to its functional impact on circuit stability and disease progression. Flavonoids such as naringenin have been shown to modulate NF-κB signaling, oxidative stress responses [118], and inflammatory mediators in zebrafish models [47,48], and may therefore serve as experimental tools to test whether fine-tuning immune reactivity can stabilize vulnerable neural circuits without disrupting physiological microglial functions. While their pleiotropic nature complicates attribution to single molecular targets [131,132,133], this characteristic may also represent an advantage, allowing graded rather than binary modulation of inflammatory pathways. Nevertheless, important limitations remain. Similarly to most flavonoids, naringenin exhibits limited oral bioavailability and undergoes extensive metabolism in humans [134,135,136,137], raising translational questions regarding dose equivalence and CNS exposure. Moreover, clinical studies often evaluate naringenin within mixed formulations rather than as an isolated compound [118], complicating interpretation. Similarly, immersion-based exposure in zebrafish does not directly replicate mammalian pharmacokinetics or BBB constraints, meaning that findings should be interpreted primarily as mechanistic insights rather than therapeutic validation. Given that most hereditary cerebellar ataxias remain without disease-modifying therapies, identifying early and potentially modifiable contributors to circuit dysfunction becomes a priority. The consistent observation that microglial activation precedes neuronal loss across multiple models [7,8,11] suggests the existence of a functional window during which neuroimmune modulation may influence disease trajectory. In this context, zebrafish models provide a complementary platform to define mechanistic thresholds of neuroimmune regulation and to prioritize candidate strategies for subsequent validation in mammalian systems.

## 9. Conclusions

A central concept emerging from this review is that neuroimmune signaling in hereditary ataxias represents a temporally sensitive process in which the timing and magnitude of modulation critically determine functional outcomes. Evidence from mammalian models indicates that inflammatory activation often precedes overt Purkinje cell degeneration, challenging the view of neuroinflammation as a purely secondary phenomenon. At the same time, the region-specific properties of cerebellar microglia and their role in synaptic refinement highlight that both excessive activation and excessive suppression can destabilize circuit integrity. In this framework, zebrafish models provide an experimentally accessible system for interrogating neuroimmune dynamics during early and pre-degenerative stages. The integration of genetic ataxia models, immune reporter lines, and pharmacological perturbation enables direct investigation of how inflammatory tone influences cerebellar circuit resilience. Flavonoids such as naringenin should therefore not be considered primarily as therapeutic endpoints, but as biologically coherent experimental probes to define functional boundaries of neuroimmune modulation in vivo. By clarifying how early modulation of inflammatory signaling shapes disease trajectory, zebrafish-based approaches may help prioritize strategies for subsequent validation in mammalian systems and support the development of future interventions for currently incurable cerebellar ataxias. Looking forward, the main opportunity offered by zebrafish models is not simply the addition of further experimental layers, but the possibility to interrogate neuroimmune function in a causally tractable and dynamic in vivo context. The combination of genetic ataxia models with established immune reporter lines already allows real-time assessment of microglial states during early and pre-degenerative stages, while pharmacological and genetic perturbations provide a means to test how changes in inflammatory tone impact cerebellar circuit stability. In this framework, the key challenge is no longer the detection of neuroinflammatory signatures, but the definition of their functional relevance for neuronal vulnerability and network dysfunction. Multi-modal readouts, including imaging and behavioral profiling, are therefore most informative when used to link microglial states to measurable circuit-level outcomes rather than as isolated descriptive endpoints. This perspective may help move the field from correlation-based descriptions of inflammation toward experimentally defined principles governing neuron–glia interactions in the cerebellum.

## Figures and Tables

**Table 1:** Main transgenic zebrafish lines for cerebellar and neuroimmune studies.

Transgenic Line/Strategy	Labeled Cell Population	Main Application	Reference(s)
aldoca (zebrafish aldolase C) Tg(aldoca:GFP) Tg(aldoca:Gal4)	Purkinje-like neurons	In vivo imaging of Purkinje cell morphology, survival, and dysfunction	[82]
Tg(-7.5ca8:GFP)bz12	Purkinje-like neurons	In vivo imaging	[85]
Tg(tagRFP-T:PC:GCaMP5G)	Purkinje-like neurons	In vivo imaging of Purkinje cell morphology, calcium imaging	[86]
Gal4/UAS cerebellar enhancer trap lines	Cerebellar neurons and Bergmann glia-like cells	Cell-type-specific labeling and functional manipulation of cerebellar circuits	[87]
Tg(gfap:GFP)	Radial glia/Bergmann glia-like cells	Analysis of neuron–glia interactions and glial responses to injury or inflammation	[83]
Tg(apoeb:GFP)	Marker for zebrafish microglia	In vivo imaging of microglia	[27]
Tg(mfap4:tdTomato-CAAX)	Microglia/macrophages	Live, Long-Term Analysis of Macrophage Behavior	[88]
Tg(mpeg1:GFP)	Microglia/macrophages	Live imaging of microglial dynamics and neuroimmune responses	[26,27,28]

**Table 2:** Representative Zebrafish models of hereditary ataxias with documented cerebellar dysfunction and ataxia-like phenotypes.

Human Disease/Gene	Zebrafish Model	Model Generation Strategy	Key Cerebellar/Motor Phenotypes Reported	Neuroimmune or Glial Features Reported	Reference(s)
SCA1-ATXN1	PC-specific expression of mutant ATXN1	Stable transgenic	Progressive Purkinje-like neuron dysfunction and degeneration; impaired exploratory behavior	Not directly assessed	[89]
SCA3-ATXN3	UAS–ATXN3[84Q]	Stable Gal4/UAS transgenic	Motor impairment; polyglutamine aggregation; functional rescue by pharmacological treatment	Not directly assessed	[94]
SCA13—*KCNC3*	kcnc3a mutant/PC-specific expression	Transgenic/targeted expression	Altered cerebellar excitability; eye-movement deficits; Purkinje-like neuron degeneration (context-dependent)	Not directly assessed	[85]
ARSACS—*SACS*	*sacs* ^−/−^	Stable CRISPR/Cas9 knockout	Impaired locomotion; cerebellar structural alterations; altered Purkinje-like neuron activity	Activation of glial cells	[95,96]
Friedreich’s ataxia—*FXN*	*fxn* loss-of-function	CRISPR/knockdown approaches	Motor impairment and cerebellar-related behavioral deficits; systemic phenotype	Oxidative stress-related pathways implicated	[97]
Spinocerebellar ataxia, autosomal recessive type 32 (SCAR32)	*prdx3* F0 crispant	CRISPR/Cas9 knockdown/F0 crispant	Motor deficits; mitochondrial dysfunction	Indirect redox–glia interaction suggested	[98]
RFC1-related ataxia (CANVAS)—*RFC1*	*rfc1* loss-of-function	CRISPR/Cas9 knockout	Impaired locomotion and balance; cerebellar-related motor dysfunction	Not directly assessed	[99]
Ataxia–telangiectasia—*ATM*	*atm* loss-of-function	CRISPR/Cas9 knockout	Locomotor deficits; neurodevelopmental abnormalities consistent with ataxia-like phenotype	Not directly assessed	[100]

**Table 3:** Experimental studies investigating naringenin in non-cerebellar zebrafish neuroinflammatory and stress-related contexts.

Context	Developmental Stage	Model Type	Main Endpoints Assessed	Principal Findings	Reference
Acute stress-induced anxiety	Adults	Acute stress exposure	Novel tank test; light–dark test (anxiety-like behaviors)	Reduced anxiety-like behavior after naringenin treatment	[125]
Neuromuscular toxicity	Larvae	BMAA exposure	Neuromuscular phenotype; myogenic and inflammatory gene expression	Restoration of structural integrity and modulation of inflammatory pathways	[47]
Neurobehavioral toxicity	Adults	BPA exposure	Novel tank test; light–dark test; oxidative stress markers	Amelioration of anxiety-like behavior and oxidative stress	[48]
Seizure model	Larvae	PTZ-induced seizures	Locomotor activity; stress-related molecular markers	Reduction in hyperactivity and modulation of stress pathways	[123]
Dopaminergic neurodegeneration	Larvae/Adults	6-OHDA exposure	Locomotor behavior; Parkinsonian gene markers	Improvement of motor phenotypes and transcriptional normalization	[126]
Alcohol-induced hepatic injury	Larvae	Ethanol exposure	Hepatic steatosis; apoptosis; lipid metabolism genes	Reduced steatosis and apoptosis; improved metabolic regulation	[127]
Hyperglycemia/retinal stress	Larvae	Glucose-induced hyperglycemia	Oxidative stress markers; VEGF expression	Decreased oxidative damage and VEGF levels	[128]

## Data Availability

No new data were created or analyzed in this study.

## Abbreviations

The following abbreviations are used in this manuscript:

BMAA	β-N-methylamino-L-alanine
BPA	Bisphenol A
PTZ	Pentylenetetrazole
6-OHDA	6-hydroxydopamine
CCe	Corpus cerebelli
Va	Valvula cerebelli
3Rs	Replacement, Reduction, and Refinement
STING	Stimulator of interferon genes
NLRP3	NOD-, LRR- and pyrin domain-containing protein 3

## References

[1] Wilson D.M., Cookson M.R., Van Den Bosch L., Zetterberg H., Holtzman D.M., Dewachter I. (2023). Hallmarks of Neurodegenerative Diseases. Cell.

[2] García-Domínguez M. (2025). Neuroinflammation: Mechanisms, Dual Roles, and Therapeutic Strategies in Neurological Disorders. Curr. Issues Mol. Biol..

[3] Bernardi E., López-Lombardía Ó., Olmedo-Saura G., Pagonabarraga J., Kulisevsky J., Pérez-Pérez J. (2026). Hereditary Ataxias: From Pathogenesis and Clinical Features to Neuroimaging, Fluid, and Digital Biomarkers—A Scoping Review. Int. J. Mol. Sci..

[4] Coarelli G., Wirth T., Tranchant C., Koenig M., Durr A., Anheim M. (2023). The Inherited Cerebellar Ataxias: An Update. J. Neurol..

[5] Jayadev S., Bird T.D. (2013). Hereditary Ataxias: Overview. Genet. Med..

[6] Mitoma H., Adhikari K., Aeschlimann D., Chattopadhyay P., Hadjivassiliou M., Hampe C.S., Honnorat J., Joubert B., Kakei S., Lee J. (2016). Consensus Paper: Neuroimmune Mechanisms of Cerebellar Ataxias. Cerebellum.

[7] Ferro A., Sheeler C., Rosa J.-G., Cvetanovic M. (2019). Role of Microglia in Ataxias. J. Mol. Biol..

[8] Aikawa T., Mogushi K., Iijima-Tsutsui K., Ishikawa K., Sakurai M., Tanaka H., Mizusawa H., Watase K. (2015). Loss of MyD88 Alters Neuroinflammatory Response and Attenuates Early Purkinje Cell Loss in a Spinocerebellar Ataxia Type 6 Mouse Model. Hum. Mol. Genet..

[9] Seki T., Sato M., Kibe Y., Ohta T., Oshima M., Konno A., Hirai H., Kurauchi Y., Hisatsune A., Katsuki H. (2018). Lysosomal Dysfunction and Early Glial Activation Are Involved in the Pathogenesis of Spinocerebellar Ataxia Type 21 Caused by Mutant Transmembrane Protein 240. Neurobiol. Dis..

[10] Shen Y., McMackin M.Z., Shan Y., Raetz A., David S., Cortopassi G. (2016). Frataxin Deficiency Promotes Excess Microglial DNA Damage and Inflammation That Is Rescued by PJ34. PLoS ONE.

[11] Quek H., Luff J., Cheung K., Kozlov S., Gatei M., Lee C.S., Bellingham M.C., Noakes P.G., Lim Y.C., Barnett N.L. (2016). A Rat Model of Ataxia-Telangiectasia: Evidence for a Neurodegenerative Phenotype. Hum. Mol. Genet..

[12] Cvetanovic M., Ingram M., Orr H., Opal P. (2015). Early Activation of Microglia and Astrocytes in Mouse Models of Spinocerebellar Ataxia Type 1. Neuroscience.

[13] Parvez M.S.A., Ohtsuki G. (2022). Acute Cerebellar Inflammation and Related Ataxia: Mechanisms and Pathophysiology. Brain Sci..

[14] Levi H., Bar E., Cohen-Adiv S., Sweitat S., Kanner S., Galron R., Mitiagin Y., Barzilai A. (2022). Dysfunction of Cerebellar Microglia in Ataxia-telangiectasia. Glia.

[15] Lai J., Demirbas D., Kim J., Jeffries A.M., Tolles A., Park J., Chittenden T.W., Buckley P.G., Yu T.W., Lodato M.A. (2024). ATM-Deficiency-Induced Microglial Activation Promotes Neurodegeneration in Ataxia-Telangiectasia. Cell Rep..

[16] Apolloni S., Milani M., D’Ambrosi N. (2022). Neuroinflammation in Friedreich’s Ataxia. Int. J. Mol. Sci..

[17] Pernaci C., Johnson A., Gillette S., Warden A.S., McCormick C., Weiser-Novak S., Ramirez G., Broersma E.H., Mishra P., Sivakumar A. (2025). Microgliopathy as a Primary Mediator of Neuronal Death in Models of Friedreich’s Ataxia. Nat. Commun..

[18] Khan W., Corben L.A., Bilal H., Vivash L., Delatycki M.B., Egan G.F., Harding I.H. (2022). Neuroinflammation in the Cerebellum and Brainstem in Friedreich Ataxia: An [^18^F]-FEMPA PET Study. Mov. Disord..

[19] Hampe C.S., Mitoma H. (2022). A Breakdown of Immune Tolerance in the Cerebellum. Brain Sci..

[20] Dukhinova M.S., Guo J., Shen E., Liu W., Huang W., Shen Y., Wang L. (2026). Cerebellar Microglia: On the Edge between Neuroinflammation and Neuroregulation. Neural Regen. Res..

[21] Xie S.-T., Fan W.-C., Zhao X.-S., Ma X.-Y., Li Z.-L., Zhao Y.-R., Yang F., Shi Y., Rong H., Cui Z.-S. (2023). Proinflammatory Activation of Microglia in the Cerebellum Hyperexcites Purkinje Cells to Trigger Ataxia. Pharmacol. Res..

[22] Yamamoto M., Kim M., Imai H., Itakura Y., Ohtsuki G. (2019). Microglia-Triggered Plasticity of Intrinsic Excitability Modulates Psychomotor Behaviors in Acute Cerebellar Inflammation. Cell Rep..

[23] Shim H.G., Jang S.-S., Kim S.H., Hwang E.M., Min J.O., Kim H.Y., Kim Y.S., Ryu C., Chung G., Kim Y. (2018). TNF-α Increases the Intrinsic Excitability of Cerebellar Purkinje Cells through Elevating Glutamate Release in Bergmann Glia. Sci. Rep..

[24] Coomey R., Stowell R., Majewska A., Tropea D. (2020). The Role of Microglia in Neurodevelopmental Disorders and Their Therapeutics. Curr. Top. Med. Chem..

[25] Mastenbroek L.J.M., Kooistra S.M., Eggen B.J.L., Prins J.R. (2024). The Role of Microglia in Early Neurodevelopment and the Effects of Maternal Immune Activation. Semin. Immunopathol..

[26] Oosterhof N., Holtman I.R., Kuil L.E., Van Der Linde H.C., Boddeke E.W.G.M., Eggen B.J.L., Van Ham T.J. (2017). Identification of a Conserved and Acute Neurodegeneration-specific Microglial Transcriptome in the Zebrafish. Glia.

[27] Peri F., Nüsslein-Volhard C. (2008). Live Imaging of Neuronal Degradation by Microglia Reveals a Role for V0-ATPase A1 in Phagosomal Fusion In Vivo. Cell.

[28] Ellett F., Pase L., Hayman J.W., Andrianopoulos A., Lieschke G.J. (2011). Mpeg1 Promoter Transgenes Direct Macrophage-Lineage Expression in Zebrafish. Blood.

[29] Mastrogiovanni M., Martínez-Navarro F.J., Bowman T.V., Cayuela M.L. (2024). Inflammation in Development and Aging: Insights from the Zebrafish Model. Int. J. Mol. Sci..

[30] Chen J., Poskanzer K.E., Freeman M.R., Monk K.R. (2020). Live-Imaging of Astrocyte Morphogenesis and Function in Zebrafish Neural Circuits. Nat. Neurosci..

[31] Gall L.G., Stains C.M., Freitas-Andrade M., Jia B.Z., Patel N., Megason S.G., Lacoste B., O’Brown N.M. (2025). Zebrafish Glial-Vascular Interactions Progressively Expand over the Course of Brain Development. iScience.

[32] Quelle-Regaldie A., Sobrido-Cameán D., Barreiro-Iglesias A., Sobrido M.J., Sánchez L. (2021). Zebrafish Models of Autosomal Recessive Ataxias. Cells.

[33] Quelle-Regaldie A., Sobrido-Cameán D., Barreiro-Iglesias A., Sobrido M.J., Sánchez L. (2021). Zebrafish Models of Autosomal Dominant Ataxias. Cells.

[34] Wang W., Gao X., Liu L., Guo S., Duan J., Xiao P. (2025). Zebrafish as a Vertebrate Model for High-Throughput Drug Toxicity Screening: Mechanisms, Novel Techniques, and Future Perspectives. J. Pharm. Anal..

[35] Strähle U., Scholz S., Geisler R., Greiner P., Hollert H., Rastegar S., Schumacher A., Selderslaghs I., Weiss C., Witters H. (2012). Zebrafish Embryos as an Alternative to Animal Experiments—A Commentary on the Definition of the Onset of Protected Life Stages in Animal Welfare Regulations. Reprod. Toxicol..

[36] Ameen-Ali K.E., Allen C., Allen C., Mocho J.-P. (2024). The 3Rs in Zebrafish Research. Zebrafish.

[37] Siddiqui S., Siddiqui H., Riguene E., Nomikos M. (2025). Zebrafish: A Versatile and Powerful Model for Biomedical Research. BioEssays.

[38] Medrano-Jiménez E., Meza-Sosa K.F., Urbán-Aragón J.A., Secundino I., Pedraza-Alva G., Pérez-Martínez L. (2022). Microglial Activation in Alzheimer’s Disease: The Role of Flavonoids and microRNAs. J. Leukoc. Biol..

[39] Jasim M.H., Saadoon Abbood R., Sanghvi G., Roopashree R., Uthirapathy S., Kashyap A., Sabarivani A., Ray S., Mustafa Y.F., Yasin H.A. (2025). Flavonoids in the Regulation of Microglial-Mediated Neuroinflammation; Focus on Fisetin, Rutin, and Quercetin. Exp. Cell Res..

[40] Dos Santos B.L., Dos Santos C.C., Soares J.R.P., Da Silva K.C., De Oliveira J.V.R., Pereira G.S., De Araújo F.M., Costa M.D.F.D., David J.M., Da Silva V.D.A. (2023). The Flavonoid Agathisflavone Directs Brain Microglia/Macrophages to a Neuroprotective Anti-Inflammatory and Antioxidant State via Regulation of NLRP3 Inflammasome. Pharmaceutics.

[41] Chen Y., Peng F., Xing Z., Chen J., Peng C., Li D. (2022). Beneficial Effects of Natural Flavonoids on Neuroinflammation. Front. Immunol..

[42] Yang G., Xu X., Gao W., Wang X., Zhao Y., Xu Y. (2025). Microglia-Orchestrated Neuroinflammation and Synaptic Remodeling: Roles of pro-Inflammatory Cytokines and Receptors in Neurodegeneration. Front. Cell. Neurosci..

[43] Zhang B., Wei Y.-Z., Wang G.-Q., Li D.-D., Shi J.-S., Zhang F. (2019). Targeting MAPK Pathways by Naringenin Modulates Microglia M1/M2 Polarization in Lipopolysaccharide-Stimulated Cultures. Front. Cell. Neurosci..

[44] Wu L.-H., Lin C., Lin H.-Y., Liu Y.-S., Wu C.Y.-J., Tsai C.-F., Chang P.-C., Yeh W.-L., Lu D.-Y. (2016). Naringenin Suppresses Neuroinflammatory Responses Through Inducing Suppressor of Cytokine Signaling 3 Expression. Mol. Neurobiol..

[45] Park H.Y., Kim G.Y., Choi Y.H. (2012). Naringenin Attenuates the Release of Pro-Inflammatory Mediators from Lipopolysaccharide-Stimulated BV2 Microglia by Inactivating Nuclear Factor-κB and Inhibiting Mitogen-Activated Protein Kinases. Int. J. Mol. Med..

[46] Rashid S.M., James A.W., Shehjar F., Yousuf S., Shah Z.A. (2026). Naringenin Ameliorates LPS-Induced Neuroinflammation Through NF-κB Signaling in Human Microglia and Protects Neuronal Cells. Brain Sci..

[47] Cao L., Zhan T., Zhao Y., Zhou J., Yang H., Zheng J., Cao B., Liu H. (2026). Naringenin Protects Zebrafish Larvae from BMAA-Induced Neuromuscular Toxicity by Regulating Myogenic and Inflammatory Pathways. Ecotoxicol. Environ. Saf..

[48] Thayumanavan G., Jeyabalan S., Fuloria S., Sekar M., Ravi M., Selvaraj L.K., Bala L., Chidambaram K., Gan S.H., Rani N.N.I.M. (2022). Silibinin and Naringenin against Bisphenol A-Induced Neurotoxicity in Zebrafish Model—Potential Flavonoid Molecules for New Drug Design, Development, and Therapy for Neurological Disorders. Molecules.

[49] Marek S., Siegel J.S., Gordon E.M., Raut R.V., Gratton C., Newbold D.J., Ortega M., Laumann T.O., Adeyemo B., Miller D.B. (2018). Spatial and Temporal Organization of the Individual Human Cerebellum. Neuron.

[50] Rudolph S., Badura A., Lutzu S., Pathak S.S., Thieme A., Verpeut J.L., Wagner M.J., Yang Y.-M., Fioravante D. (2023). Cognitive-Affective Functions of the Cerebellum. J. Neurosci..

[51] Michell-Robinson M.A., Touil H., Healy L.M., Owen D.R., Durafourt B.A., Bar-Or A., Antel J.P., Moore C.S. (2015). Roles of Microglia in Brain Development, Tissue Maintenance and Repair. Brain J. Neurol..

[52] La Sala G., Farini D. (2025). Glial Cells and Aging: From the CNS to the Cerebellum. Int. J. Mol. Sci..

[53] Stowell R.D., Wong E.L., Batchelor H.N., Mendes M.S., Lamantia C.E., Whitelaw B.S., Majewska A.K. (2018). Cerebellar Microglia Are Dynamically Unique and Survey Purkinje Neurons In Vivo. Dev. Neurobiol..

[54] Grabert K., Michoel T., Karavolos M.H., Clohisey S., Baillie J.K., Stevens M.P., Freeman T.C., Summers K.M., McColl B.W. (2016). Microglial Brain Region−dependent Diversity and Selective Regional Sensitivities to Aging. Nat. Neurosci..

[55] Perez-Pouchoulen M., VanRyzin J.W., McCarthy M.M. (2015). Morphological and Phagocytic Profile of Microglia in the Developing Rat Cerebellum. eneuro.

[56] Ayata P., Badimon A., Strasburger H.J., Duff M.K., Montgomery S.E., Loh Y.-H.E., Ebert A., Pimenova A.A., Ramirez B.R., Chan A.T. (2018). Epigenetic Regulation of Brain Region-Specific Microglia Clearance Activity. Nat. Neurosci..

[57] Leung A.W., Li J.Y.H. (2018). The Molecular Pathway Regulating Bergmann Glia and Folia Generation in the Cerebellum. Cerebellum.

[58] Witter L., Rudolph S., Pressler R.T., Lahlaf S.I., Regehr W.G. (2016). Purkinje Cell Collaterals Enable Output Signals from the Cerebellar Cortex to Feed Back to Purkinje Cells and Interneurons. Neuron.

[59] Millen K.J. (2025). Essential New Insights into Human Cerebellar Purkinje Cell Biology from Studies of Essential Tremor. Proc. Natl. Acad. Sci. USA.

[60] Millen K.J., Gleeson J.G. (2008). Cerebellar Development and Disease. Curr. Opin. Neurobiol..

[61] Bellamy T.C. (2006). Interactions between Purkinje Neurones and Bergmann Glia. Cerebellum.

[62] Hikosaka M., Parvez M.S.A., Yamawaki Y., Oe S., Liang Y., Wada Y., Hirahara Y., Koike T., Imai H., Oishi N. (2025). Maternal Immune Activation Followed by Peripubertal Stress Combinedly Produce Reactive Microglia and Confine Cerebellar Cognition. Commun. Biol..

[63] Manto M., Marmolino D. (2009). Cerebellar Disorders—At the Crossroad of Molecular Pathways and Diagnosis. Cerebellum.

[64] Deus C.M., Tavares H., Beatriz M., Mota S., Lopes C. (2022). Mitochondrial Damage-Associated Molecular Patterns Content in Extracellular Vesicles Promotes Early Inflammation in Neurodegenerative Disorders. Cells.

[65] Yang K., Dunn M., Torres-Ramirez G., Dobbs N., Shakkottai V.G., Yan N. (2025). Autonomous STING Signaling in Purkinje Cells Drives Neurodegeneration Independent of Type I Interferon. Cell Rep..

[66] Fujita Y., Yamashita T. (2021). Alterations in Chromatin Structure and Function in the Microglia. Front. Cell Dev. Biol..

[67] Wang L., Yu C.-C., Liu X.-Y., Deng X.-N., Tian Q., Du Y.-J. (2021). Epigenetic Modulation of Microglia Function and Phenotypes in Neurodegenerative Diseases. Neural Plast..

[68] Vilca S.J., Margetts A.V., Pollock T.A., Tuesta L.M. (2023). Transcriptional and Epigenetic Regulation of Microglia in Substance Use Disorders. Mol. Cell. Neurosci..

[69] Ferro A., Qu W., Lukowicz A., Svedberg D., Johnson A., Cvetanovic M. (2018). Inhibition of NF-κB Signaling in IKKβF/F;LysM Cre Mice Causes Motor Deficits but Does Not Alter Pathogenesis of Spinocerebellar Ataxia Type 1. PLoS ONE.

[70] Pansera L., Mhalhel K., Cavallaro M., Aragona M., Laurà R., Levanti M., Guerrera M.C., Abbate F., Germanà A., Montalbano G. (2025). Zebrafish as an Integrative Model for Central Nervous System Research: Current Advances and Translational Perspectives. Life.

[71] Huang H., Lindgren A., Wu X., Liu N.-A., Lin S. (2012). High-Throughput Screening for Bioactive Molecules Using Primary Cell Culture of Transgenic Zebrafish Embryos. Cell Rep..

[72] Lubin A., Otterstrom J., Hoade Y., Bjedov I., Stead E., Whelan M., Gestri G., Paran Y., Payne E. (2021). A Versatile, Automated and High-Throughput Drug Screening Platform for Zebrafish Embryos. Biol. Open.

[73] Auer F., Nardone K., Matsuda K., Hibi M., Schoppik D. (2025). Cerebellar Purkinje Cells Control Posture in Larval Zebrafish (*Danio rerio*). Elife.

[74] Pose-Méndez S., Schramm P., Valishetti K., Köster R.W. (2023). Development, Circuitry, and Function of the Zebrafish Cerebellum. Cell. Mol. Life Sci..

[75] Hashimoto M., Hibi M. (2012). Development and Evolution of Cerebellar Neural Circuits. Dev. Growth Differ..

[76] Namikawa K., Dorigo A., Köster R.W. (2019). Neurological Disease Modelling for Spinocerebellar Ataxia Using Zebrafish. J. Exp. Neurosci..

[77] Hibi M., Shimizu T. (2012). Development of the Cerebellum and Cerebellar Neural Circuits. Dev. Neurobiol..

[78] Jurisch-Yaksi N., Yaksi E., Kizil C. (2020). Radial Glia in the Zebrafish Brain: Functional, Structural, and Physiological Comparison with the Mammalian Glia. Glia.

[79] Barber H.M., Robbins C.G., Cutler Z., Brown R.I., Jung L.E., Werkman I., Kucenas S. (2025). Radial Astroglia Cooperate with Microglia to Clear Neuronal Cell Bodies during Zebrafish Optic Tectum Development. Cell Rep..

[80] Oosterhof N., Boddeke E., Van Ham T.J. (2015). Immune Cell Dynamics in the CNS: Learning from the Zebrafish. Glia.

[81] Li Y., Chen T., Miao X., Yi X., Wang X., Zhao H., Lee S.M.-Y., Zheng Y. (2017). Zebrafish: A Promising in Vivo Model for Assessing the Delivery of Natural Products, Fluorescence Dyes and Drugs across the Blood-Brain Barrier. Pharmacol. Res..

[82] Koyama W., Hosomi R., Matsuda K., Kawakami K., Hibi M., Shimizu T. (2021). Involvement of Cerebellar Neural Circuits in Active Avoidance Conditioning in Zebrafish. eneuro.

[83] Bernardos R.L., Raymond P.A. (2006). GFAP Transgenic Zebrafish. Gene Expr. Patterns.

[84] Mazzolini J., Chia K., Sieger D. (2018). Isolation and RNA Extraction of Neurons, Macrophages and Microglia from Larval Zebrafish Brains. J. Vis. Exp..

[85] Namikawa K., Dorigo A., Zagrebelsky M., Russo G., Kirmann T., Fahr W., Dübel S., Korte M., Köster R.W. (2019). Modeling Neurodegenerative Spinocerebellar Ataxia Type 13 in Zebrafish Using a Purkinje Neuron Specific Tunable Coexpression System. J. Neurosci..

[86] Matsui H., Namikawa K., Babaryka A., Köster R.W. (2014). Functional Regionalization of the Teleost Cerebellum Analyzed In Vivo. Proc. Natl. Acad. Sci. USA.

[87] Takeuchi M., Matsuda K., Yamaguchi S., Asakawa K., Miyasaka N., Lal P., Yoshihara Y., Koga A., Kawakami K., Shimizu T. (2015). Establishment of Gal4 Transgenic Zebrafish Lines for Analysis of Development of Cerebellar Neural Circuitry. Dev. Biol..

[88] Walton E.M., Cronan M.R., Beerman R.W., Tobin D.M. (2015). The Macrophage-Specific Promoter Mfap4 Allows Live, Long-Term Analysis of Macrophage Behavior during Mycobacterial Infection in Zebrafish. PLoS ONE.

[89] Namikawa K., Pose-Méndez S., Köster R.W. (2024). Genetic Modeling of Degenerative Diseases and Mechanisms of Neuronal Regeneration in the Zebrafish Cerebellum. Cell. Mol. Life Sci..

[90] Sellier C., Campanari M., Julie Corbier C., Gaucherot A., Kolb-Cheynel I., Oulad-Abdelghani M., Ruffenach F., Page A., Ciura S., Kabashi E. (2016). Loss of C9ORF72 Impairs Autophagy and Synergizes with polyQ Ataxin-2 to Induce Motor Neuron Dysfunction and Cell Death. EMBO J..

[91] Yanicostas C., Barbieri E., Hibi M., Brice A., Stevanin G., Soussi-Yanicostas N. (2012). Requirement for Zebrafish Ataxin-7 in Differentiation of Photoreceptors and Cerebellar Neurons. PLoS ONE.

[92] Low S.E., Woods I.G., Lachance M., Ryan J., Schier A.F., Saint-Amant L. (2012). Touch Responsiveness in Zebrafish Requires Voltage-Gated Calcium Channel 2.1b. J. Neurophysiol..

[93] Wen H., Linhoff M.W., Hubbard J.M., Nelson N.R., Stensland D., Dallman J., Mandel G., Brehm P. (2013). Zebrafish Calls for Reinterpretation for the Roles of P/Q Calcium Channels in Neuromuscular Transmission. J. Neurosci..

[94] Watchon M., Yuan K.C., Mackovski N., Svahn A.J., Cole N.J., Goldsbury C., Rinkwitz S., Becker T.S., Nicholson G.A., Laird A.S. (2017). Calpain Inhibition Is Protective in Machado–Joseph Disease Zebrafish Due to Induction of Autophagy. J. Neurosci..

[95] Naef V., Damiani D., Licitra R., Marchese M., Della Vecchia S., Baggiani M., Brogi L., Galatolo D., Landi S., Santorelli F.M. (2025). Modeling Sacsin Depletion in Danio Rerio Offers New Insight on Retinal Defects in ARSACS. Neurobiol. Dis..

[96] Naef V., Marchese M., Ogi A., Fichi G., Galatolo D., Licitra R., Doccini S., Verri T., Argenton F., Morani F. (2021). Efficient Neuroprotective Rescue of Sacsin-Related Disease Phenotypes in Zebrafish. Int. J. Mol. Sci..

[97] Rojsajjakul T., Do W., Wilson R.B., Blair I.A. (2025). Stable Isotope Labeling in Bacteria Enables Characterization and Quantification of Frataxin Protein in a Friedreich’s Ataxia Zebrafish Model. Anal. Chem..

[98] Naef V., Lieto M., Satolli S., De Micco R., Troisi M., Pasquariello R., Doccini S., Privitera F., Filla A., Tessitore A. (2024). SCAR32: Functional Characterization and Expansion of the Clinical-genetic Spectrum. Ann. Clin. Transl. Neurol..

[99] Nobilleau F., Audet S., Da Silva Babinet A., Tork S., Zaouter C., Liao M., Pilon N., Tétreault M., Patten S.A., Samarut É. (2025). RFC1 Regulates the Expansion of Neural Progenitors in the Developing Zebrafish Cerebellum. Nat. Commun..

[100] Chen K., Wang P., Chen J., Ying Y., Chen Y., Gilson E., Lu Y., Ye J. (2022). Loss of Atm in Zebrafish as a Model of Ataxia–Telangiectasia Syndrome. Biomedicines.

[101] Panche A.N., Diwan A.D., Chandra S.R. (2016). Flavonoids: An Overview. J. Nutr. Sci..

[102] Aggarwal D., Chaudhary M., Mandotra S.K., Tuli H.S., Chauhan R., Joshi N.C., Kaur D., Dufossé L., Chauhan A. (2025). Anti-Inflammatory Potential of Quercetin: From Chemistry and Mechanistic Insight to Nanoformulations. Curr. Res. Pharmacol. Drug Discov..

[103] Herrera T.E.S., Tello I.P.S., Mustafa M.A., Jamil N.Y., Alaraj M., Atiyah Altameem K.K., Alasheqi M.Q., Hamoody A.-H.M., Alkhafaji A.T., Shakir M.N. (2025). Kaempferol: Unveiling Its Anti-Inflammatory Properties for Therapeutic Innovation. Cytokine.

[104] Liu Y., Shi X., Tian Y., Zhai S., Liu Y., Xiong Z., Chu S. (2023). An Insight into Novel Therapeutic Potentials of Taxifolin. Front. Pharmacol..

[105] Jang S., Johnson R.W. (2010). Can Consuming Flavonoids Restore Old Microglia to Their Youthful State?. Nutr. Rev..

[106] Pecorini G., Votta A., Tiralongo G., Volpi D., Ferraro E., Puppi D. (2024). Naringenin-Loaded Poly(3-Hydroxybutyrate-Co-3-Hydroxyvalerate)-Based Devices Have an Anti-Inflammatory Activity on Microglia. J. Drug Deliv. Sci. Technol..

[107] Khanna S., Kumar S., Sharma P., Daksh R., Nandakumar K., Shenoy R.R. (2025). Flavonoids Regulating NLRP3 Inflammasome: A Promising Approach in Alleviating Diabetic Peripheral Neuropathy. Inflammopharmacology.

[108] Spencer J.P.E., Vafeiadou K., Williams R.J., Vauzour D. (2012). Neuroinflammation: Modulation by Flavonoids and Mechanisms of Action. Mol. Asp. Med..

[109] Al-Khayri J.M., Sahana G.R., Nagella P., Joseph B.V., Alessa F.M., Al-Mssallem M.Q. (2022). Flavonoids as Potential Anti-Inflammatory Molecules: A Review. Molecules.

[110] Abbate F., Maugeri A., Laurà R., Levanti M., Navarra M., Cirmi S., Germanà A. (2021). Zebrafish as a Useful Model to Study Oxidative Stress-Linked Disorders: Focus on Flavonoids. Antioxidants.

[111] Mhalhel K., Sicari M., Pansera L., Chen J., Levanti M., Diotel N., Rastegar S., Germanà A., Montalbano G. (2023). Zebrafish: A Model Deciphering the Impact of Flavonoids on Neurodegenerative Disorders. Cells.

[112] Solanki I., Parihar P., Mansuri M.L., Parihar M.S. (2015). Flavonoid-Based Therapies in the Early Management of Neurodegenerative Diseases. Adv. Nutr..

[113] Vantaggiato C., Guarato G., Brivio F., Panzeri E., Speltoni B., Gumeni S., Orso G., Santorelli F.M., Bassi M.T. (2025). Naringenin and SMER28 Target Lysosomal Reformation and Rescue SPG11 and SPG15 Hereditary Spastic Paraplegia Phenotypes. Pharmacol. Res..

[114] Napoli B., Gumeni S., Forgiarini A., Fantin M., De Filippis C., Panzeri E., Vantaggiato C., Orso G. (2019). Naringenin Ameliorates Drosophila ReepA Hereditary Spastic Paraplegia-Linked Phenotypes. Front. Neurosci..

[115] Escribano-Ferrer E., Queralt Regué J., Garcia-Sala X., Boix Montañés A., Lamuela-Raventos R.M. (2019). In Vivo Anti-Inflammatory and Antiallergic Activity of Pure Naringenin, Naringenin Chalcone, and Quercetin in Mice. J. Nat. Prod..

[116] Lai Z., Ke L., Zhao W. (2025). Naringenin as a Neurotherapeutic Agent in Alzheimer’s Disease: Epigenetic Signatures, Gut Microbiota Alterations, and Molecular Neuroprotection. Front. Aging Neurosci..

[117] Zhu Y., Guo X., Li S., Wu Y., Zhu F., Qin C., Zhang Q., Yang Y. (2024). Naringenin Ameliorates Amyloid-β Pathology and Neuroinflammation in Alzheimer’s Disease. Commun. Biol..

[118] Salehi B., Fokou P.V.T., Sharifi-Rad M., Zucca P., Pezzani R., Martins N., Sharifi-Rad J. (2019). The Therapeutic Potential of Naringenin: A Review of Clinical Trials. Pharmaceuticals.

[119] Li L., Liu R., He J., Li J., Guo J., Chen Y., Ji K. (2022). Naringin Regulates Microglia BV-2 Activation and Inflammation via the JAK/STAT3 Pathway. Evid.-Based Complement. Altern. Med..

[120] Piano I., Votta A., Colucci P., Corsi F., Vitolo S., Cerri C., Puppi D., Lai M., Maya-Vetencourt J.F., Leigheb M. (2023). Anti-Inflammatory Reprogramming of Microglia Cells by Metabolic Modulators to Counteract Neurodegeneration; a New Role for Ranolazine. Sci. Rep..

[121] Khan M.B., Khan M.M., Khan A., Ahmed M.E., Ishrat T., Tabassum R., Vaibhav K., Ahmad A., Islam F. (2012). Naringenin Ameliorates Alzheimer’s Disease (AD)-Type Neurodegeneration with Cognitive Impairment (AD-TNDCI) Caused by the Intracerebroventricular-Streptozotocin in Rat Model. Neurochem. Int..

[122] Dai X.-J., Jia Y., Cao R., Zhou M.-N. (2023). Naringin Prevents Cognitive Dysfunction in Aging Rats by Inhibiting Toll-Like Receptor 4 (TLR4)/NF-κB Pathway and Endoplasmic Reticulum Stress. Evid.-Based Complement. Altern. Med..

[123] Manish R., Brindha D., Deenathayalan U. (2024). Behavioral Rescue: Naringenin’s Neuroprotective Effects against PTZ-Induced Seizures by Mitigating Oxidative Stress and Neuroinflammation in Zebrafish Larvae. Pharmacol. Res.-Mod. Chin. Med..

[124] Xu S., Wu B., Zhong B., Lin L., Ding Y., Jin X., Huang Z., Lin M., Wu H., Xu D. (2021). Naringenin Alleviates Myocardial Ischemia/Reperfusion Injury by Regulating the Nuclear Factor-Erythroid Factor 2-Related Factor 2 (Nrf2)/System Xc-/Glutathione Peroxidase 4 (GPX4) Axis to Inhibit Ferroptosis. Bioengineered.

[125] Nachammai V., Jeyabalan S., Muthusamy S. (2021). Anxiolytic Effects of Silibinin and Naringenin on Zebrafish Model: A Preclinical Study. Indian J. Pharmacol..

[126] Kesh S., Kannan R.R., Balakrishnan A. (2021). Naringenin Alleviates 6-Hydroxydopamine Induced Parkinsonism in SHSY5Y Cells and Zebrafish Model. Comp. Biochem. Physiol. Part C Toxicol. Pharmacol..

[127] Lin H., Zhou Z., Zhong W., Huang P., Ma N., Zhang Y., Zhou C., Lai Y., Huang S., An H. (2017). Naringenin Inhibits Alcoholic Injury by Improving Lipid Metabolism and Reducing Apoptosis in Zebrafish Larvae. Oncol. Rep..

[128] Sudhakaran G., Chandran A., Sreekutty A.R., Madesh S., Pachaiappan R., Almutairi B.O., Arokiyaraj S., Kari Z.A., Tellez-Isaias G., Guru A. (2023). Ophthalmic Intervention of Naringenin Decreases Vascular Endothelial Growth Factor by Counteracting Oxidative Stress and Cellular Damage in In Vivo Zebrafish. Molecules.

[129] Nakayama H., Abe M., Morimoto C., Iida T., Okabe S., Sakimura K., Hashimoto K. (2018). Microglia Permit Climbing Fiber Elimination by Promoting GABAergic Inhibition in the Developing Cerebellum. Nat. Commun..

[130] Mu Y., Bennett D.V., Rubinov M., Narayan S., Yang C.-T., Tanimoto M., Mensh B.D., Looger L.L., Ahrens M.B. (2019). Glia Accumulate Evidence That Actions Are Futile and Suppress Unsuccessful Behavior. Cell.

[131] Boesten D.M.P.H.J., Von Ungern-Sternberg S.N.I., Den Hartog G.J.M., Bast A. (2015). Protective Pleiotropic Effect of Flavonoids on NAD^+^ Levels in Endothelial Cells Exposed to High Glucose. Oxidative Med. Cell. Longev..

[132] Stachelska M.A., Karpiński P., Kruszewski B. (2025). A Comprehensive Review of Biological Properties of Flavonoids and Their Role in the Prevention of Metabolic, Cancer and Neurodegenerative Diseases. Appl. Sci..

[133] Punmiya A., Prabhu A. (2023). Structural Fingerprinting of Pleiotropic Flavonoids for Multifaceted Alzheimer’s Disease. Neurochem. Int..

[134] Berga M., Logviss K., Lauberte L., Paulausks A., Mohylyuk V. (2023). Flavonoids in the Spotlight: Bridging the Gap between Physicochemical Properties and Formulation Strategies. Pharmaceuticals.

[135] Peng Y., Qu R., Xu S., Bi H., Guo D. (2024). Regulatory Mechanism and Therapeutic Potentials of Naringin against Inflammatory Disorders. Heliyon.

[136] Jia Y., Zhou X., Liu Y., Liu X., Ren F., Liu H. (2025). Novel Insights Into Naringenin: A Multifaceted Exploration of Production, Synthesis, Health Effects, Nanodelivery Systems, and Molecular Simulation. Mol. Nutr. Food Res..

[137] Flores-Peña R., Monroy-Ramirez H.C., Caloca-Camarena F., Arceo-Orozco S., Salto-Sevilla J.A., Galicia-Moreno M., Armendariz-Borunda J. (2025). Naringin and Naringenin in Liver Health: A Review of Molecular and Epigenetic Mechanisms and Emerging Therapeutic Strategies. Antioxidants.

